# Does Using Highly Porous Tantalum in Revision Total Hip Arthroplasty Reduce the Rate of Periprosthetic Joint Infection? A Systematic Review and Meta-Analysis

**DOI:** 10.1016/j.artd.2023.101293

**Published:** 2024-01-17

**Authors:** Peyman Mirghaderi, Nasim Eshraghi, Erfan Sheikhbahaei, Mohammadreza Razzaghof, Kiarash Roustai-Geraylow, Alireza Pouramini, Mohammad Mirahmadi Eraghi, Fatemeh Kafi, Sayed Mohammad Javad Mortazavi

**Affiliations:** a Surgical Research Society (SRS), Students’ Scientific Research Center, Tehran University of Medical Sciences, Tehran, Iran; b Joint Reconstruction Research Center (JRRC), Tehran University of Medical Sciences, Tehran, Iran

**Keywords:** Arthroplasty, Hip, Prosthesis-related infections, PJI, Tantalum

## Abstract

**Background:**

Studies suggest tantalum (Ta) implants may have inherent antibacterial properties. However, there is no consensus regarding the effectiveness of Ta in preventing periprosthetic joint infection (PJI) after revision total hip arthroplasty (rTHA).

**Methods:**

We searched 5 main databases for articles reporting the rate of PJI following rTHA using Ta implants from inception to February 2022. The PJI rates of the Ta group were meta-analyzed, compared with the control group, and represented as relative risks (RRs) in forest plots.

**Results:**

We identified 67 eligible studies (28,414 joints) for assessing the prevalence of PJI following rTHA using Ta implants. Among these studies, only 9 compared the Ta implant group with a control group. The overall PJI rate following rTHA using Ta implants was 2.9% (95% confidence interval [CI]: 2.2%-3.8%), while it was 5.7% (95% CI = 4.1%-7.8%) if only septic revisions were considered. Comparing the Ta and control groups showed a significantly lower PJI rate following all-cause rTHA with an RR = 0.80 (95% CI = 0.65-0.98, *P* < .05). There was a trend toward lower reinfection rates in the Ta group after rTHA in septic cases, although the difference was not statistically significant (RR = 0.75, 95% CI = 0.44-1.29, *P* = .30).

**Conclusions:**

Ta implants are associated with a lower PJI rate following all-cause rTHA but not after septic causes. Despite positive results, the clinical significance of Ta still remains unclear since the PJI rate was only reduced by 20%.

**Level of Evidence:**

IV.

## Introduction

Periprosthetic joint infection (PJI) is the most unwelcome complication following a total hip arthroplasty (THA) [1,2]. It is associated with significant morbidity and mortality and poses a substantial economic burden of as much as $753.4 million on the healthcare system annually [3,4]. However, PJI following a revision THA (rTHA) is more frequent and far more burdensome [5]. A recent large-sample study in the Medicare population found PJI as the most common complication of rTHA, with an incidence of 17.3% at 1-year follow-up. The rate of new PJI after aseptic rTHA was 8.13% [6]. Moreover, the recurrence rate of PJI after a one-stage rTHA was reported to be between 6.5% and 33% [7,8].

The increase in the aging population and hip osteoarthritis prevalence has caused a dramatic rise in THA rates [9]. Consequently, the rates of rTHA have been predicted to double from 2005-2026 [10]. Moreover, data from UK National Joint Registry has shown a 3-fold increase in re-revision rates due to PJI from 2005-2013 [5]. Given the expected surge in rates of rTHA, it is prudent to seek strategies to reduce the rates of post-rTHA PJIs.

Trabecular metal (TM) components with highly porous tantalum (Ta) coatings are one of these strategies. Ta’s superior outcomes in rTHA are mainly attributed to its high porosity promoting osteointegration, similar elastic modulus to trabecular bone reducing the stress shielding, and good frictional profile decreasing micromotion [11]. It has also been reported to be more resistant to infection than titanium. Tokarski et al found a significantly lower rate of post-rTHA PJI using Ta (3.1%) than titanium (17.5%) [12]. Hypothetically, its higher and faster osteointegration leaves less dead space for bacteria to grow, its 3D structure hinders the formation of a biofilm compared with a flat surface, and its chemistry has been proposed to be hostile to bacteria [12,13]. Moreover, an in vitro study showed lower *S. aureus* adhesion to pure Ta than titanium, while another similar study failed to show any difference [14,15]. Similarly, the in vivo efficacy and inherent antimicrobial potential of Ta are uncertain [13,15]

The 2018 International Consensus Meeting on PJI in Philadelphia (ICM Philly) tried to answer whether Ta can reduce the risk of PJI recurrence in rTHA. Although their answer was positive, especially for the treatment of PJIs, the evidence was limited [16]. In addition, they suggested that Ta augments may protect against PJI recurrence following a single-stage revision for PJI [17]. Apart from the limited evidence of both statements, it is still unclear whether the potential effects of highly porous Ta differ in rTHAs for septic vs aseptic reasons. Therefore, we performed this systematic review to answer the following questions: 1. What is the rate of PJI after rTHA using Ta implants? 2. Is the rate of PJI different after rTHA using Ta vs non-Ta implants? 3. Does Ta reduce the risk of PJI following rTHA for septic reasons?

## Material and methods

### Patients (P), intervention (I), comparison (C), and outcome (O)

This study examined the deep infection (PJI) rate (O) in patients undergoing rTHA (P) using TM implants containing highly porous Ta (I) compared to other materials (C).

### Screening and search strategy

We performed this study in accordance with Preferred Reporting Items for Systematic Reviews and Meta-Analyses guidelines [18] [see Additional file 1]. It was registered in the International Prospective Register of Systematic Reviews (PROSPERO, registration ID: CRD42021268518, available at www.crd.york.ac.uk/PROSPERO).

We systematically searched for clinical studies reporting the rate of PJI following rTHA using Ta implants in MEDLINE/PubMed, EMBASE, SCOPUS, Web of Science, and Cochrane Library from inception to February 2022. Our main search strategy was “(Tantalum OR Trabecular metal) AND (Infection OR PJI)”. The detailed version of our search strategy in PubMed is presented in (Table 1). Based on the search rules of each database, the search query was modified. We also performed an additional hand search of the related bibliography. The Covidence online systematic review software was used to import all the records (https://www.covidence.org). Two reviewers (A.P. and F.K.) independently reviewed the imported articles for eligibility using the inclusion/exclusion criteria in 2 steps: title/abstract and full-text screening. Any conflict was resolved by discussion and consultation with a third reviewer (P.M).

**Table 1:** PubMed search strategy (using [all fields] and [MeSH]).

Component 1 (infection)		Component 2 (tantalum)
"Prosthesis-Related Infections"[MeSH] OR Prosthetic∗ Infection∗ OR "Surgical Wound Infection"[MeSH] OR Surgical Wound Infection∗ OR Surgical Site Infection∗ OR Periprosthetic joint infection∗ OR Postoperative Wound Infection∗ OR PJI OR SSI OR Infect∗ OR Septic∗ OR "Sepsis"[MeSH] OR Sepsis∗ OR "Anti-Infective Agents"[MeSH] OR Anti-Infective OR Anti Infective OR Anti-infective OR "Anti-Bacterial Agents"[MeSH] OR Microbicide∗ OR Bacteriocide∗ OR Anti-Microb∗ OR Anti Microb∗ OR AntiMicrob∗ OR AntiBacterial∗ OR Anti-Bacterial∗ OR Antibacterial∗ OR Postoperative Complication∗ OR "Postoperative Complications"[MeSH] OR "Long Term Adverse Effects"[MeSH] OR Long Term Adverse Effect∗	AND	"Tantalum"[MeSH] OR Tantalum∗ OR Trabecular metal OR ∗

### Inclusion and exclusion criteria

We included the original studies reporting the rate of PJI following rTHA using Ta implants. The exclusion criteria were: 1) those studies excluding PJIs; 2) non-English studies; 3) nonhuman studies; 4) reviews, congress abstracts, and book chapters; and 5) studies with a follow-up duration of less than 3 months.

### Assessment of study quality

Two independent reviewers assessed the quality of the included studies based on the Newcastle-Ottawa Scale (NOS) [19]. It has 8 items in 3 categories of selection, comparability of the study groups, and the outcome assessment. Total score can range between 0 and 9. Those studies with 7 or more points were of high, and those with less than 4 points were of low quality.

### Data extraction

The study data were extracted and organized into a predesigned Excel sheet (Microsoft Office 2016). The data included demographics, study groups, population, revision indication (septic vs aseptic), re-revision rate, PJI rate in septic and aseptic cases, and implant details.

### Statistical analysis and data synthesis

After article selection, a meta-analysis was conducted on the applicable outcome measures using the Comprehensive Meta-analysis software (version 3). We calculated the pooled infection rate following rTHA with Ta implants based on all included studies. From this selection, we specifically chose studies that compared the Ta implant group with a control group to calculate the relative risk (RR). A meta-analysis of the RR of the studies with control groups was done. The funnel plot and Egger’s test were used to analyze publication bias. Cochran's Q and I^2^ tests were used to identify heterogeneities among the included studies. When the Q and degrees of freedom (Q/df) ratio is less than one, heterogeneity is nonsignificant. I^2^ values of 25%, 50%, and 75% revealed low, moderate, and high heterogeneity, respectively [20]. When I^2^ was greater than 50%, the random-effects model was used. Sensitivity analysis was used to find the sources of heterogeneity. If the same authors reported a newer study from the same population, only the latest study would be included in the analysis. Moreover, studies with fewer than 10 patients as the denominator of prevalence were excluded from the analysis. The *P*-value < .05 was considered significant.

## Results

### Included studies and quality assessment

**Figure 1:**
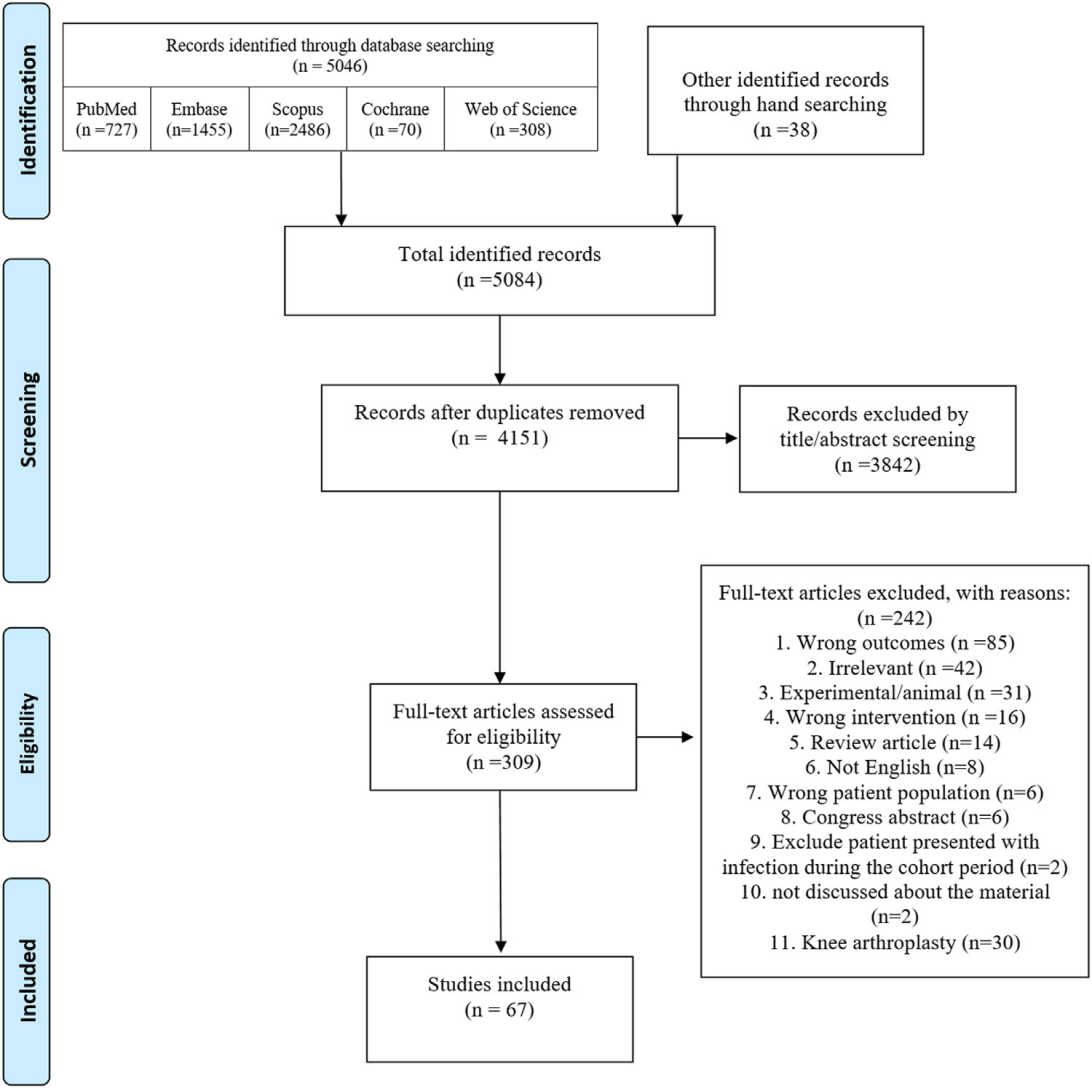
Preferred Reporting Items for Systematic Reviews and Meta-Analyses diagram of the studies' selection process.

A total of 4151 studies were screened by title and abstract after removing the duplicates (Fig. 1). Finally, 67 articles were selected after full-text evaluation for eligibility. Table 2 provides a summary of the 67 rTHA studies (28,414 hips) showing the studies' characteristics and outcomes [12,[21], [22], [23], [24], [25], [26], [27], [28], [29], [30], [31], [32], [33], [34], [35], [36], [37], [38], [39], [40], [41], [42], [43], [44], [45], [46], [47], [48], [49], [50], [51], [52], [53], [54], [55], [56], [57], [58], [59], [60], [61], [62], [63], [64], [65], [66], [67], [68], [69], [70], [71], [72], [73], [74], [75], [76], [77], [78], [79], [80], [82], [83], [84]]. The study quality assessed by NOS was high in 31 (46.3%), medium in 27 (40.3%), and low in 9 (13.4%) studies (Supplementary Table). The mean (standard deviation) NOS score was 6.3 ± 1.7. Overall, the studies showed medium to high quality. All 9 studies with control groups were of high quality according to the NOS assessment. However, 4 out of 9 (44%) disclosed conflicts of interest, while the same number (44%) revealed funding from companies or institutions.

**Table 2:** Summary of the rTHA included studies' characteristics and outcomes.

No.	Study author and year	Study design	Group(s)	Population (hips)	Revision indication	Follow-up (months, ±SD or range)	Total re-revision rate	Infection rate (PJI)	Subsequent infection (recurrence of PJI)	Conclusion about effectiveness of tantalum in terms of infection	Tantalum details
1	Russell 2021 [67]	Prospective cohort	TM augments for severe acetabular defects	38	Aseptic loosening = 34 (89.5%) Two-stage revision for deep infection = 4 (10.5%)	7.3 y (5.4-10.8)	7 (18.4%)	3 (7.9%)	3 (75%)	This long-term study details our experience of TM augments for the most severe acetabular defects. For such cases, no excellent surgical solution exists; in comparison to alternative methods, we advocate that this technique is reasonably safe and effective	TM augments
2	Cassar-Gheiti 2021 [21]	Retrospective cohort	Use of flying buttress augments in revision THA	59	Aseptic loosening = 39 (66.1%) Infection= 9 (15.3%) Instability = 4 (6.8%) Osteolysis = 4 (6.8%) Periprosthetic fracture = 1 (1.7%) Component malposition = 1 (1.7%) Pseudotumor = 1 (1.7%)	8 y (2-17)	5 (8.5%)	4 (6.7%)	-	Treatment of superolateral acetabular defects during revision THA using porous Ta augments placed in the type I (flying buttress) configuration provides excellent implant survivorship and favorable clinical outcomes at mid-term follow-up.	TM augments
3	Xiao 2021 [22]	Retrospective cohort	Acetabular revision with porous Ta augments and titanium-coated cups	41	Aseptic loosening = 33 Periprosthetic infection = 8	122.8 (69-165)	0 (0%)	0 (0%)	0 (0%)	The combination of Ta augments and conventional titanium-coated cups achieved satisfactory long-term radiographic and clinical outcomes for Paprosky type III acetabular bone defects without pelvic discontinuity.	Ta augment
4	Bawale 2021 [23]	Retrospective cohort	TM Ta modular uncemented cup	59 hips (58 patients)	Aseptic loosening = 35 (60.3%) Femoral loosening = 7 (11.8%) Fracture = 7 (11.8%) Cup Mal-alignment = 5 (8.8%) Infection = 2 (3.3%) Metal-on-metal related pathology = 3 (4%)	87 (24-144)	2 (3.2%)	1 (1.6%)	-	The use of Ta acetabular components during revision THA is associated with a lower incidence of infection (infection rate of 1.6% for deep infection)	Ta cup
5	Simon 2021 [24]	Case report	TM augment for proximal metaphyseal femoral defect in rTHA	3	Osteolysis = 1 Periprosthetic fracture = 1 PJI = 1	15.8	0	0	-	For severe proximal femur bone defects, Ta cones may be used for metaphyseal bone reconstruction.	TM augment
6	Miettinen 2020 [68]	Retrospective cohort	Ta metal acetabulum component	100	Loosening of acetabulum = 67 linear wearing = 19 loosening of both components = 5 infection = 2 semiendoprothesis penetration through acetabulum = 2 component malposition with pain = 2 Periprosthetic fracture = 1 Loosening femur = 1 Recurrent dislocation = 1	11.5 ± 4.118 y	18 (18%)	6 (6%)		The TM acetabular component gives excellent outcomes regarding stability and fixation to the acetabulum in acetabulum revision hip arthroplasty at a minimum of 10 y of follow-up. However, acetabular component malposition and the small head size (28 mm) are risk factors for dislocation.	TM acetabular component
7	Baecker 2020 [25]	Retrospective cohort	Ta augments combined with antiprotrusio cages	20	Aseptic cup loosening = 14 (70%) PJI = 6 (30%)	2.8 y (2-5.8)	5 (25%)	2 (10%)	2 (33.3%)	In summary, a Ta augment combined with an antiprotrusio cage in Paprosky IIIA and IIIB defects with divergent anatomy not amenable to a hemispherical socket provides a reliable technique to restore the anatomic hip center and prevent superior migration and provides a bony ingrowth surface. Longer term follow-up is required before the technique is widely adapted.	Ta augment
8	Chiarlone 2020 [26]	Retrospective cohort	Revision THA with Ta in severe bone defect eg, Paprosky III A–B	9	Acetabular aseptic loosening = 5 (55.6%) Osteolysis = 3 (33.3%) Second-stage reimplantation for PJI= 1 (11.1%)	35.3 ± 10.8 (minimum: 2-y)	3 (33.3%)	1 (11.1%)	-	NA	Double porous Ta cup
9	Chacko 2020 [27]	Retrospective cohort	High friction TM sockets	146	ARMD = 55 (37.7%) Instability/Dislocation = 12 (8.2%) Psoas impingement = 3 (2.1%) Periprosthetic fracture = 11 (7.5%) Infection = 20 (13.7%)	43.5 (25-62)	6 (4.2%)	2 (1.37%)	-	NA	TM acetabular component
10	Zhang 2020 [28]	Retrospective cohort	Double-TM cups for revision of complex acetabular defects	18 hip (18 patients)	Aseptic loosening (100%)	61.0 (IQR: 56.0-65.8)	0 (0%)	0 (0%)	NA	Acetabular revision with double- TM cups with or without impacting bone grafting are practical and acceptable treatment	Double-TM cups
11	Cruz-Pardos 2020 [29]	Retrospective cohort	Titanium and TM cups in revision surgery for acetabular bone loss	197		8.1 y (1-15)				There was no significant difference in re-revision due to aseptic loosening or radiological loosening between titanium and TM cups in revision surgery for acetabular bone loss.	TM cup
			Group1: TM cup	116	Aseptic cup loosening = 91 (78.4%) Revision due to infection (2nd stage) = 3 (2.6%) Polyethylene wear = 6 (5.2%) Other = 16 (13.8%)	5.4 ± 3.1 y	5 (4.3%), *P* = .54	0 (0%)	0 (0%)		
			Group2: Ti cup	81	Aseptic cup loosening = 53 (65.4%) Revision due to infection (2nd stage) = 5 (6.2%) Polyethylene wear = 4 (4.9%) Other = 19 (23.5%)	12.0 ± 7.8 y	2 (2.5%)	0 (0%)	0 (0%)		
12	Theil 2019 [69]	Retrospective cohort	Porous Ta revision cups	41	Aseptic loosening = 34 (83%) Periprosthetic joint infection= 7 (17%)	72	10 (27%)	2 (4.9%)	1 (14.3%)	The use of porous Ta metal implants in acetabular revision surgery achieves good to excellent short- term and mid-term functional results and an acceptable complication rate relative to the extent of defect and previous surgery. However, one should be aware of potential limitations of the implants in addressing large defects and discontinuity.	Acetabular component and augment
13	Ebied 2019 [30]	Prospective cohort	Using a combination of Ta metal augments (TMAs) and impaction graft in single-stage revision for periprosthetic infection	48		5 y (2-7)				Metal augments can convert massive acetabular defects to a more contained defect suitable for grafting. The combination of Ta augments that provide strong structural support and antibiotic-loaded allograft is successful in the mid-term in single-stage revisions for infection.	Ta augments
			Group1: Ta augments for acetabular wall reconstruction	22	Infection N = 22		1 (4.5%)	1 (4.5%)	1 (4.5%)		
			Group2: Control group not received metal augments	26	Infection N = 26		1 (3.8%)	1 (3.8%)	1 (3.8%)		
14	Löchel 2019 [71]	Retrospective cohort	Porous Ta shells and augments	53	Aseptic loosening = 49 Revision for infection = 4	10 y	4 (7.5%)	1 (1.9%)	-	The reconstruction of acetabular defects with TM shells and augments showed excellent long-term results. Supplementary screw fixation of the shell should be performed in every patient. Alternative techniques should be considered to address pelvic discontinuity.	TM shell and augment
15	Matharu 2019 [31]	Retrospective matched cohort	Only revisions due to the infections	Total: 794 hips (722 patients)	PJI	5.3 y (1.0-13.5)	34 (4.3%)	42 (5.3%)	42 (5.3%)	No significant difference in the all-cause risk of acetabular re-revision was detected when comparing the TM population with the non-TM population following THA revision due to the PJI TM implant does not reduce the recurrence of infection rate	TM cup
			Group1: TM cups = 541	TM = 541	PJI		21 (3.9%)	28 (5.2%) (Sub hazard ratio, 0.70; 95% CI, 0.29-1.69; *P* = .427)	28 (5.2%)		
			Group2: Non-TM cups = 253	Non-TM = 253	PJI		14 (5.5%)	14 (5.5%)	14 (5.5%)		
16	Cursaru 2019 [85]	Retrospective cohort	Ta augments for major acetabular bone defects in revision THA	11 hip (11 patients)	-	23 (11-36)	0 (0%)	0 (0%)	-	NA	Ta Augments
17	Li 2019 [32]	Retrospective cohort	Trabecular metal cup and augments	16 hips (18 patients)	Aseptic loosening = 15 Septic loosening = 3	27.72 ± 12.18 mo	0 (0%)	0 (0%)	-	There was a statistically significant correlation between 3D planned and postoperative value. Preoperative 3D simulation and model were considered the useful method to assist implant positioning in complex revision THA, with moderate to high accuracy, and with satisfied clinical outcome and lower complication rate. Moreover, it had high accuracy in predicting number and size of TM augments used intraoperatively.	TM cup and augments
18	Brüggemann 2018 [33]	Retrospective cohort	Porous Ta shells	184 Dual-mobility cups = 69 Polyethylene liners = 115	Polyethylene liners: Loosening = 98 (53%) Dislocation = 3 (1%) Infection = 6 (2%) Other = 8 (4%) Dual-mobility cups: Loosening N = 56 (30%) Dislocation N = 2 (1%) Infection N= 6 (3%) Other N = 5 (2%)	4.9 (0.5-8.9) y	17 (9.2%)	1 (0.05%)	0 (0%)	Cementing a Dual-mobility cups into a TM shell provide a promising therapeutic choice in acetabular revision surgery	Ta shells + Augments
19	O'Neill 2018 [34]	Retrospective cohort	TM Augments	38	Aseptic loosening = 34 Infection (2-stage revision) = 4	36 (18-74)	3 (7.9%) Revision for: Aseptic = 2 Sepsis = 1	2 (5.3%)	NA	Establishing TM at complex acetabular reconstruction yield promising outcomes in the short-medium following up	Ta shells + Augments
20	Matharu 2018 [35]	Retrospective cohort	TM coated acetabular components	Total = 11,988	ARMD = 1415 Infection= 802 Fracture = 634 Loosening/lysis = 6758 Dislocation/subluxation = 763 Other = 1151 Unexplained pain = 465	6.1 y (1.0-12.7)	302 (2.5%)	141 (1.2%)	14 (5.7%, out of 247 hips)	Six-year rates of re-revision for infection was comparable between revision total hip arthroplasties with TM and non-TM coatings. TM-coated acetabular components was in company with a low risk of septic re-revision, which was comparable with non-TM components	Ta acetabular components
			Group1: TM	8497	ARMD = 1284 Infection= 549 Fracture = 460 Loosening/lysis = 4580 Dislocation/subluxation = 508 Other = 848 Unexplained pain = 268		2.5%	1.1%	7 (6.03%, out of 116 hips)		
			Group2: Non-TM	3491	ARMD = 131 Infection= 253 Fracture = 174 Loosening/lysis = 2178 Dislocation/subluxation = 255 Other = 303 Unexplained pain = 465		2.6%	1.4%	7 (5.34%, out of 131 hips)		
21	Eachempati 2018 [36]	Retrospective cohort	TM augments in Paprosky IIIA defect AND Paprosky IIIB defect	41 hips (41 patients)	Aseptic loosening with IIIA and IIIB defects	39.4 (12-96)	0 (0%)	0 (0%)	NA	TM augments with cementless TM for revision acetabular components is contributed to excellent outcomes in patients suffering Paprosky IIIA and IIIB acetabular defects.	Porous TM augments And shell
22	Loppini 2018 [37]	Retrospective cohort	TM double- cup in revision of Paprosky type III defects	16 hips (16 patients)	Aseptic loosening	34 (24-72)	0 (0%)	0 (0%)	NA	NA	Double-TM cup
23	Chang 2018 [38]	Retrospective cohort	Structural allograft accompanied by a TM-coated hemispherical cup for Paprosky type III defects	20	Paprosky type III defects	5.4 y (3.3-10.3)	0 (0%)	0 (0%)	-	Mid-term clinical results for treatment of Paprosky type III acetabular defects with a structural allograft and Ta TM cup were satisfactory	TM-coated cup
24	Lachiewicz 2018 [39]	Retrospective cohort	Acetabular revision using a TM acetabular component	48	Acetabular component loosening = 34 Recurrent dislocation = 4 Osteolysis = 5 Two-stage revisions = 4 Bipolar endoprosthesis with protrusion = 1	5 y (3-7)	5 (10.4%)	1 (2.1%)	-	TM components appear to provide durable fixation at midterm followup in complex acetabular revisions. Steps to minimize dislocation, the most frequent complication of these revisions, may include the routine use of larger femoral heads.	Ta acetabular component
25	López 2018 [40]	Retrospective cohort		84		4.77 y (2-7.5)	10 (11.9%)	3 (3.6%)	0 (0%)	No significant differences in the frequency of infection were observed between the 2 implant systems.	TM augment
			Group1: TM system	58	Aseptic loosening = 43 (74.1%) Septic loosening = 9 (15.5%) Acetabular fracture = 3 (5.2%) Acetabular erosion = 1 (1.7%) Instability = 2 (3.4%)		5 (8.6%) *P* = .16	7 (12.1%) *P* = .94	0 (0%)		
			Group2: Burch-Schneider antiprotrusion cage	26	Aseptic loosening = 18 (69.2%) Septic loosening = 6 (23.1%) Acetabular fracture = 0 Acetabular erosion = 1 (3.8%) Instability = 1 (3.8%)		5 (19.2%)	3 (11.5%)	0 (0%)		
26	Laaksonen 2017 [41]	Retrospective case-control	Porous Ta cups Vs other cementless designs in revision THA	Total = 6843	Loosening = 3958 Fracture = 265 Dislocation = 717 Infection = 272 Metal disorder = 723 Pain = 119 Other = 489		624 (9.1%)	157 (2.3%)	13 (4.8%)	No benefit in survival with re-revision for infection as the endpoint could be ascribed to the porous Ta cup group, as has been suggested by earlier work. HR for infection induced re-revision= 0.9 (0.7-1.3) *P* = .66	Porous Ta cups and augments
			Group 1: Porous Ta	2442	Loosening = 1679 Fracture = 105 Dislocation = 210 Infection = 85 Metal disorder = 157 Pain = 26 Other = 180	3.0 y (0-9)	215 (9%)	51 (2.1%)			
			Group 2: Uncemented cup	4401	Aseptic loosening = 2279 Infection = 187 Fracture = 160 Dislocation = 507 Metal-related disorder = 866 Pain = 93 Others = 309	3.4 (0-9) y	409 (9%)	106 (2.4%)			
27	Prieto 2017 [42]	Retrospective cohort	TM revision with structural allograft	58 hips (56 patients)	Aseptic loosening = 42 (73%) Loosening in relation with Pelvic discontinuity = 12 (20%), Infection (second-stage) = 4 (7%).	5.4 y (2-12)	7 (12%)	1 (1.7%)	-	TM shells combined with structural allograft in revision THA was found to achieve excellent midterm survival, with a 5-year survival ship of 94% of acetabular components acquiring stable union onto host bone.	Modular porous metal shells, augments and cup
28	Evola 2017 [86]	Retrospective cohort	Acetabular component	58	Aseptic loosening = 49 (84.5) Polyethylene wear = 7 (12.1) Infection = 2 (3.4)	87.6 ± 25.6 (range: 3-120)	3 (5%)	1 (1%)	-	The medium-term use of prosthetic Ta components in prosthetic hip revisions is safe and effective in a wide variety of acetabular bone defects.	Augment usage among
29	Brüggemann 2017 [43]	Prospective cohort	Porous Ta cups vs Müller acetabular roof reinforcement rings (MARRs)	Total: 207	Loosening = 169 Dislocation = 6 Infection = 10 Other = 22		23 (11.1%)	1 (0.5%)	-	TM cups yield favorable therapeutic outcomes in acetabular revision surgery	Porous Ta cup
			Group 1: Porous Ta cup	Porous Ta cup = 111	Loosening = 93 (84%) Dislocation = 3 (3%) Infection= 5 (5%) Other = 10 (9%)	4.9 y (2.1-6.9)	14 (12.6%)	0 (0%)	-		
			Group 2: MARRs	MARRs = 96	Loosening = 76 (79%) Dislocation = 5 (5%) Infection= 5 (3%) Other = 12 (12%)	12 y (2.3-17)	9 (9.4%)	1 (1%)	-		
30	Jenkins 2017 [44]	Retrospective cohort	Porous Ta augments	85	Paprosky type 2A: N = 4 Paprosky type 2B: N = 3 Paprosky type 2C: N = 1 Paprosky type 3A: N = 28 Paprosky type 3B: N = 22	8 (2-17)	2 (2.35%)	0 (0%)	-	This cohort demonstrated 97% survivorship and maintained satisfactory hip function at the minimum 5 y after the index revision surgery.	Porous Ta augments
31	Vutescu 2017 [45]	Retrospective cohort		Total: 796	-	Minimum 2-y	10 (1.3%)	9 (1.1%)	-	After adjusting for acetabular defect severity, both PoTa and PoTi acetabular components had excellent survival at mean 44.4-month (range 4.3-91.5 mo) follow-up when used in primary and revision THA	
			Group 1: Porous Ta cup	159	-	62.3 (25.3-91.5)	1 (0.6%)	3 (1.9%)	-		
			Group 2: Porous Ti cup	637	-	48.3 (4.3-89.4)	9 (1.4%)	6 (0.94%)	-		
32	Jeong 2016 [87]	Retrospective cohort	Ta Augment in patients with Paprosky III or IV acetabular bone defects	15	Aseptic loosening = 12 Deep infection = 3	29 (24-48)	1 (6%)	1 (6%)	-	The present study showed satisfactory clinical and radiographic outcomes of revision THA using Ta augments due to severe acetabular bone defects of Paprosky type III or IV at a minimum follow-up of 2 y.	Ta augments
33	Rowan 2016 [46]	Retrospective case-control	Comparison of acetabular impaction grafting (AIBG) and trabecular metal for revision arthroplasty	Total: 53	Osteolysis = 44 Instability = 1 Sepsis = 7 Periprosthetic fracture (femur) = 1					AIBG and TM acetabular defect reconstructions achieve good clinical outcome but there is greater success with TM in higher grades of acetabular deficiency regardless of prior infection	Ta TM acetabular component alone or Ta TM acetabular component with a Ta augment were used
			Group 1: Porous TM	17	Osteolysis = 12 Instability = 1 Sepsis = 3 Periprosthetic fracture (femur) = 1	5.4 (0.8-10.4)	0 (0%)	0 (0%)	0 (0%)		
			Group 2: AIBG	36	Osteolysis = 32 Instability = 0 Sepsis = 4 Periprosthetic fracture (femur) = 0	5.9 (0.7-12.0)	4 (11%)	3 (8%)	0 (0%)		
34	Flecher 2016 [47]	Retrospective cohort	Porous Ta components	51	Paprosky type 2A = 18 Paprosky type 2B = 11 Paprosky type 2C = 10 Paprosky type 3A = 7 Paprosky type 3B = 5	6.8 (5.1-10)	1 (1.9%)	1 (1.9%)	-	When facing an acetabular revision with severe bone loss, Ta-made components can provide a stable fixation.	TM acetabular component with a modular acetabular augment
35	Konan 2016 [48]	Retrospective cohort	Porous Ta uncemented acetabular components in revision THA	46 hips (46 patients)	Aseptic loosening with pelvic osteolysis = 37 Pelvic discontinuity = 4 Aseptic loosening secondary to failure of bony ingrowth = 2 Failure of a reconstruction cage = 2 Failed hip hemiarthroplasty with acetabular erosion = 1	11 y (10-12)	4 (9%)	0 (0%)	-	Porous Ta uncemented acetabular components yield 96% survival with shell revision in long-term follow up	Porous Ta uncemented acetabular components
36	Tokarski 2015 [12]	Retrospective case-control	All patients	990 hips (966 patients)	Loosening = 470 (47%) Instability = 120 (12%) Infection = 144 (15%) Polyethylene wear = 114 (12%) Other = 114 (14%)	40.2 (3 mo-13.1 y)	73 (7.1%)	40 (4%)	16/144 (11.1%)	Ta components possibly protect failure of revision THA from infection, especially in the subgroup of patients with prior PJI	Acetabular component
			Group 1: Ta acetabular components	454 hips	Loosening = 235 Instability = 33 Infection = 64 Polyethylene wear = 48 Other = 74		20 (4.4%) *P* < .001	13 (2.9%) *P* = .105	2/64 (3.1%) *P* = .007		
			Group 2: Ti acetabular components	536 hips	Loosening = 235 Instability = 87 Infection = 80 Polyethylene wear = 66 Other = 68		53 (9.9%)	27 (5%)	14/80 (17.5%)		
37	Long 2015 [88]	Retrospective cohort	Porous Ta acetabular components	599	Instability = 24 Septic joint = 14	38 (24-72)	40 (6.7%)	14 (2.33%)	-	Early results of porous Ta acetabular components in the revision setting demonstrate good initial stability and low reoperation rates at 2 y follow-u	Uncemented porous Ta
38	Mohaddes 2015 [49]	Retrospective cohort	TM cup vs non-TM cup	Total: 2460 hips (2384 patients)	-					Primary diagnosis, had no significant potentiality to affect incidence of the reversion/re-revision	TM cup
			Group 1: TM cup	TM = 805	-	3.2 y (0-7)	38 (4.7%)	11 (1.2%)	-		
			Group 2: Trilogy or Lubinus cup	N = 1655	-		94 (5.7%)	41 (2.5%) RR = 0.85 0 (95% CI 0.61-1.2 *P* = .3)	-		
39	Whitehouse 2015 [50]	Retrospective cohort	Acetabular TM augments. Primary: N = 3 Revision: N= 53	40	-	110 (88-128)	4 (10%)	1 (2.5%)	-	In the long term, TM implants continue to yield encouraging results with low rates of revision or loosening in this complex group of patients.	Modular TM Augments
40	Callado 2014 [51]	Retrospective cohort	TM wedge in patients with a minimum grading of Paprosky II-B	23	-	29.5	1 (4.1%)	1 (4.1%)	-	NA	Acetabular component TM wedge (Augments)
41	Munro 2014 [52]	Retrospective cohort	Porous Ta shell	15	Adverse local tissue reactions = 19 Deep infection = 3 Acetabular components’ loosening = 10	25 (10-48)	-	0 (0%)	0 (0%)	NA	Porous Ta shell
42	Klatte 2014 [89]	Retrospective case–control	Ta augments in the setting of one-stage exchange of infected THA	97	PJI = 97					Ta augments are a viable option for addressing acetabular defects in one-stage exchange for septic THA, and does not show a higher risk for a reinfection rate in comparison to the single-stage control group without using Ta augments	TM acetabular augments
			Group1: Porous Ta acetabular augments and a cemented acetabular cup system	50	PJI = 50	3 y (1.5-4.5)	8 (16%)	2 (4%)	2 (4%)		
			Group2: Without the use of trabecular augments	47	PJI = 47	3 y (1.6-4.1)	10 (21%)	2 (4.2%)	2 (4.2%)		
43	Batuyong 2014 [90]	Retrospective cohort	Porous Ta Acetabular Components	24	Aseptic loosening = 18 (75%) Osteolysis = 2 (8%) Instability = 2 (8%) Infection= 1 (4%) Failure of hemiarthroplasty = 1 (4%)	37 ± 14 (range, 24-66)	5 (20.8%)	3 (12.5%)	-	When dealing with severe acetabular bone loss, porous Ta acetabular components show promising short-term results.	
44	Moličnik 2014 [53]	Retrospective cohort	TM acetabular Component	25	Aseptic loosening = 22 (88%) catastrophic liner wear + metallosis+ periacetabular pseudotumor = 2 (8%) Chronic luxation following multiple revisions = 1 (4%)	20.9 (12-42)	1 (4%)	0 (0%)	-	NA	TM acetabular shell with or without augments+ Nonmodular shells
45	Abolghasemian 2013 [91]	Retrospective cohort	Combined TM acetabular shell and augment	34	Aseptic loosening = 29 Failure of a cage = 1 Two-stage revision for infected THA = 2 Previous resection arthroplasty for infection = 2	64.5 (27-107)	5 (14%)	2 (1.9%)	-	Good clinical and radiological results can be expected for bone-deficient acetabula treated by a TM cup and augment, but for pelvic discontinuities this might not be a reliable option	TM acetabular shell and one or 2 augments
46	Elganzoury 2013 [54]	Prospective cohort	Revision of acetabular components using TM cups and augments for acetabular reconstruction	18	Aseptic loosening of acetabular component	18 (12-24)	0 (0%)	0 (0%)	-	TM acetabular components and augments for acetabular defects (Paprosky II and III) appear to be a promising solution for this complex situation.	TM cups and augments
47	Sporer 2012 [92]	Retrospective cohort	Acetabular distraction technique with porous Ta components	20	Aseptic loosening	4.5 y (2-7)	1 (5%)	0 (0%)	-	Acetabular distraction with porous Ta components provides predictable pain relief and durability at 2- to 7-year follow-up when reconstructing severe acetabular defects with an associated pelvic discontinuity.	Porous Ta acetabular component alone or + modular porous Ta augment
48	Del Gaizo 2012 [55]	Retrospective cohort	Acetabular revision for the Paprosky type IIIA acetabular defects with the porous Ta	37 hips (36 patients)	Aseptic loosening of the acetabular component = 31 Infection = 5 Chronically dislocated hemi resurfacing arthroplasty = 1	60 (26-106)	7 (19%)	3 (8.33%)	-	Considerable function in company with low incidence of loosening amid the follow-up.	Porous Ta acetabular component and augment or shell
49	Borland 2012 [93]	Prospective cohort	Complex reconstruction of the acetabulum using a TM augment, impaction bone grafting, and a cemented high-density polyethylene cup	24	Massive aseptic acetabular bone loss = 24	61 (32-81)	1 (4.2%)	0 (0%)	-	TM augments are effective in filling the bone defect and provide a stable foundation for impaction bone grafting	TM augments
50	Pierannunzii 2011 [80]	Retrospective cohort	TM cup without augments	21	Aseptic loosening = 17 Infection = 3 Recurrent dislocation = 1	20.9 (13-30)	2 (9.52%)	1 (4.76%)	-	The mid-term results of this series confirm the hypothesis that a porous Ta acetabular cup is an effective option to deal with difficult acetabular revisions. Although no extra-acetabular fixation device is available, the very high surface friction guaranteed by the material and the supplemental stability provided by trans-acetabular screws seem to be sufficient to allow satisfactory reimplantation even in severely damaged pelves	TMT multi-hole acetabular cup without augmentation
51	Skyttä 2011 [56]	Retrospective cohort	Acetabular component revision using TM Revision Shell	827	Aseptic loosening = 330 (40%) Deep prosthetic infection = 39 (5%) Dislocation = 88 (11%) Socket exchange = 42 (5%) Other indications = 112 (14%).	1.1 y	40 (4.8%)	2 (0.24%)	-	We found no differences in survival rates between aseptic and septic revisions. Furthermore, sex, diagnosis, and hospital volume did not affect the survival.	TM Revision Shells
52	Davies 2011 [57]	Retrospective cohort	TM acetabular components	46	Osteolysis = 9 (20%) Aseptic loosening = 27 (59%) Infection = 4 (8%) Other = 6 (13%)	50 (28-76)	1 (2.2%)	1 (2.2%)	-	Porous Ta shows promising results in revision arthroplasty with severe acetabular bone loss.	TM acetabular component and augment
53	Ballester Alfaro 2010 [81]	Retrospective cohort	TM buttress augment and the TM cup-cage	19	Major bone loss	26 (18-43)	0 (0%)	1 (5.3%)	0 (0%)	Our early results suggest that buttress Ta augments, with cup-cage construct for severe bone defects, may be an alternative to other treatment options, but a longer follow-up is necessary.	Buttress Ta augments or cup-cage construct combined with a TM shell
54	Flecher 2010 [58]	Retrospective cohort	Tantalum implants	72	Septic acetabular loosening = 6 (8.3%)	4 y (2-6)	3 (4.2%)	1 (1.4%)	-	Ta components provide a stable primary cementless fixation without the need of a structural graft.	Tantalum cups, some with augments
55	Lachiewicz 2010 [59] New report in 2018 [39]	Prospective cohort	Acetabular revision with Ta acetabular components	39	Acetabular revisions using a Ta acetabular component for high risk patients: 37 patients with Type 3 Paprosky Two patients with weights of greater than 81 kg	3.3 y (2-7)	4 (10.3%)	0 (0%)	-	Ta acetabular components provide stable fixation in difficult acetabular revisions.	Ta acetabular component
56	Fernández-Fairen 2010 [96]	Retrospective cohort	Revision of a failed acetabular component in which TM acetabular components	263	Aseptic loosening = 186 (70.7%) Polyethylene wear = 62 (23.5%) Femoroacetabular instability/impingement = 15 (5.7%)	73.6 (60-84)	2 (0.76%)	2 (0.76%)	-	The acetabular component used was reliable in creating a durable composite without failure for a minimum of 5 y.	TM acetabular components
57	Lakstein 2009 [97]	Prospective cohort	TM cups in revision for Acetabular Defects with 50% or less contact with native bone	53 hips (53 patients)	Aseptic loosening: N = 48 (91%) Instability: N = 3 (5%) Infection: N= 2 (4%)	45 (24-71)	5 (9.4%)	0 (0%)	0 (0%)	Managing the massive contained acetabular defects with 50% or Less host bone via TM cups represents a reasonable choice.	TM cups
58	Lingaraj 2009 [60]	Retrospective cohort	Paprosky type 3 bone defect using modular porous Ta components	24 hips (23 patients)	Aseptic loosening = 21 Infection(second-stage) = 2 Periprosthetic fracture = 1	41 (24-62)	2 (8.7%)	0 (0%)	NA	Reconstruction of Paprosky type 3 acetabular defects through porous metal components yields viable outcomes	Ta acetabular components and augment
59	Siegmeth 2009 [98]	Prospective cohort	Modular Ta Augments	34	Aseptic loosening = 28 Infection = 2 mechanical loosening = 1 failed structural allograft = 1 recurrent dislocation = 1	34 (24-55)	2 (5%)	0 (0%)	0 (0)%	Of the 32 patients who had not been revised, all had stable cups radiographically. All quality-of-life parameters improved. The early results with Ta augments are promising but longer followup is required	TM augment combined with a TM shell
60	Xu 2009 [99]	Retrospective cohort	Porous Ta uncemented acetabular cup	16	-	24 (18-25)	0 (0%)	0 (0%)	-	or cases of failed primary fixation of artificial acetabular cup, application of porous Ta uncemented acetabular cup can produces favorable results in acetabular revision if no bone defects exist.	Porous Ta uncemented acetabular cup
61	Van Kleunen 2009 [61]	Retrospective cohort	Revision THA using a TM acetabular component	97 hips (90 patients)	Aseptic loosening = 73 Infection = 17 periprosthetic fracture = 2 failure of a cage construct = 2 catastrophic polyethylene liner wear = 2 periacetabular giant cell tumor = 1	45 (24-79)	13 (13.4%)	10 (10.3%)	4 (23.5%)	TM acetabular cups and shells with or without the use of modular augments can be effectively used to revise failed acetabular components in patients with substantial pelvic bone loss.	TM revision acetabular component with or without modular augments
62	Flecher 2008 [62]	Retrospective cohort	Ta cups and augments for type III defect	23 hips (22 patients)	Aseptic loosening = 17	35 (24-50)	0 (0%)	0 (0%)	-	Trabecular metal components appear suitable to achieve primary stability in type III acetabular defect as an alternative to bone graft and cages.	Ta cups and augments
63	Kim 2008 [63]	Retrospective cohort	Porous Ta uncemented acetabular shells	46	Aseptic loosening with pelvic osteolysis = 37 Pelvic discontinuity = 4 Aseptic loosening secondary to failure of bony ingrowth = 2 Failed reconstruction cage = 2 Failed hip hemiarthroplasty = 1	40 (24-51)	3 (6.5%)	0 (0%)	-	In cases of acetabular revision arthroplasties associated with severe acetabular defects, use of the porous Ta uncemented acetabular shell demonstrated promising clinical and radiographic results over a period of 2 to 4 y.	Porous Ta uncemented acetabular shells
64	Weeden 2007 [83]	Retrospective cohort	Acetabular revisions in Paprosky IIIA and IIIB treated with Ta acetabular implants	43	Aseptic loosening = 37 Sepsis = 4 Pain after a previous bipolar hemiarthroplasty = 2	2.8 y (2-4)	1 (2%)	1 (2%)	-	Implants made from highly porous Ta metal provide a surface that is highly conducive to bone ingrowth. Combined with the ability to use modular augments for added support and stability, this technology may change the way major defects are reconstructed.	Ta acetabular cups + tantalum acetabular modular augments
65	Sporer 2006 [64]	Retrospective cohort	Acetabular Revision Using a TM Acetabular Component for Severe Acetabular Bone Loss	13	-	2.6 y (1-3)	0 (0%)	0 (0%)	-	Using a TM acetabular component for the treatment of pelvic discontinuity during acetabular revision demonstrates promising short-term results	Ta acetabular component alone or + with a Ta augment
66	Sporer 2006 [65]	Retrospective cohort	TM Acetabular Component and Augment for Severe Acetabular Defects	28	Aseptic loosening = 23 Second-stage reimplantation for infection = 4 traumatic injury in = 1	3.1 y (1-4)	1 (3%)	0 (0%)	0 (0%)	The combination of TM acetabular component and a TM augment provides promising short-term results for Paprosky type IIIa acetabular defects	Ta porous coated acetabular component + Ta augment
67	Unger 2005 [66]	Retrospective cohort	Porous Tantalum Uncemented Acetabular Cup in Revision THA	60	Osteolysis/unstable = 33 (55%) Osteolysis/stable = 17 (28%) Unstable = 6 (10%) Chronic dislocation = 1 (2%) Infection = 1 (2%) Other = 2 (3%)	42 (14-68)	1 (1%)	0 (0%)	0 (0%)	TM acetabular cup provides good results in the THA revision but further studies are required.	Uncemented TM Monoblock Acetabular Cup

### Meta-analysis results

Matharu et al [31], Mohaddes et al [49], and Flecher et al [58,62] studies were excluded from the meta-analysis since more recent reports were available from their patients or databases [35,41,47]. The quantitative syntheses are summarized in (Table 3).

**Table 3:** Summary of quantitative synthesis.

Variable	Pooled effect size	95% CI	*P*-value	Model	I^2^	Q/df	Publication bias (Egger’s *P*-value)	Related figures
Infection rate after rTHA with tantalum	Rate = 2.9%	2.2%-3.8%	-	Random effect	58.7	2.4	Yes (.02^a^)	Figures 2 and 3a
Comparing the infection rate between tantalum and the control group after rTHA	RR = 0.80	0.65-0.99	.04^a^	Fixed effect	0.0	0.5	No (.99)	Figures 3b and 4
Reinfection rate after rTHA in septic cases	Rate = 5.7%	4.1%-7.8%	-	Fixed effect	47.6	1.9	No (.86)	Figures 3c and 5
Comparing the reinfection rate between tantalum and the control group after rTHA in septic cases	RR = 0.75	0.44-1.29	.30	Fixed effect	32.3	1.5	No (.68)	Figures 3d and 3

CI, confidence interval; rTHA, revision total hip arthroplasty; RR, relative risk.

a Statistically significant *P*-value.

#### PJI rate after rTHA

**Figure 2:**
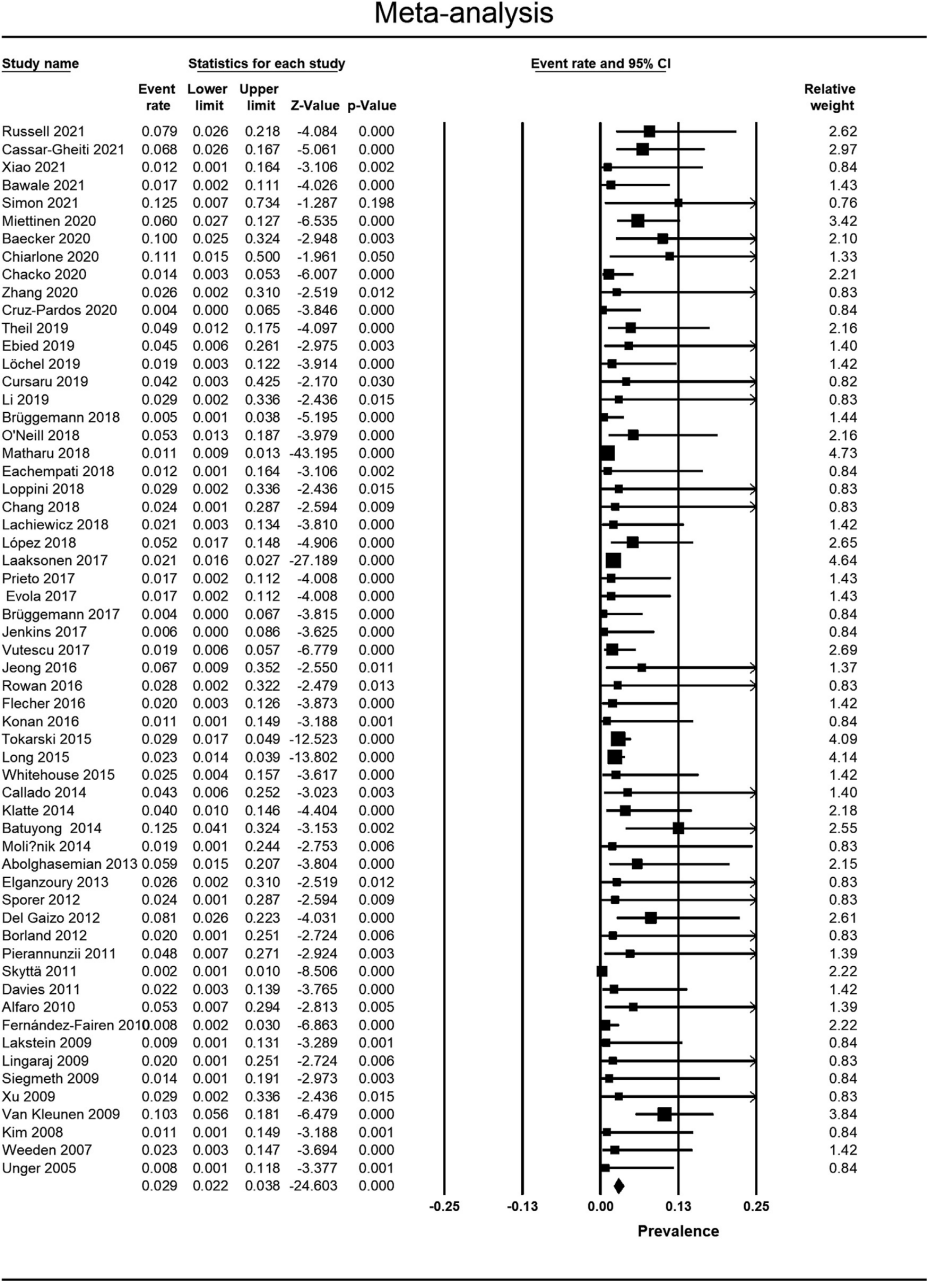
Pooled rate of infection after all-cause rTHA with Ta.

**Figure 3:**
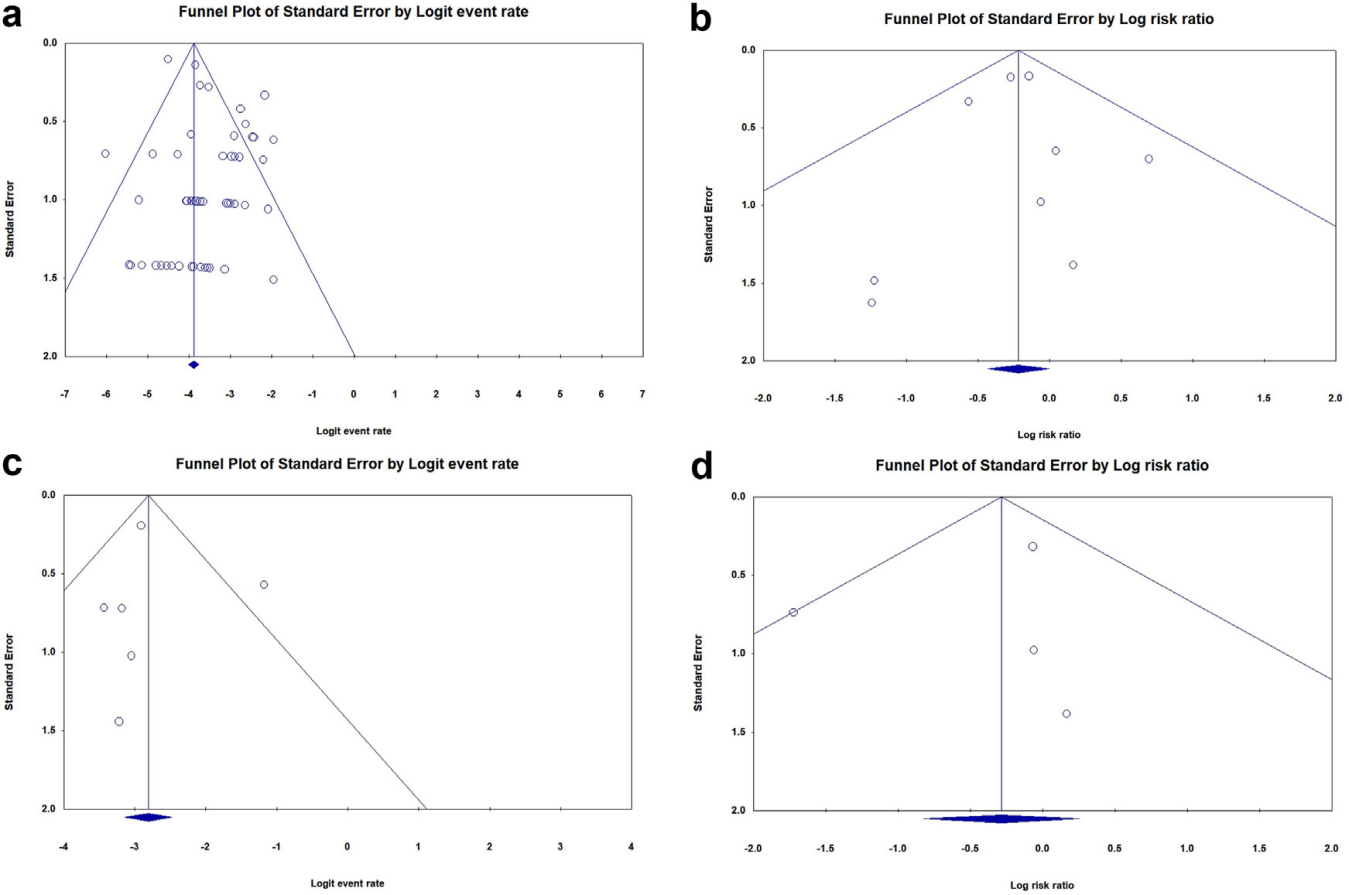
Publication bias using a funnel plot. (a) Infection rate after rTHA with tantalum, (b) comparing the infection rate between tantalum and the control group after rTHA, (c) re-infection rate after rTHA in septic cases, (d) comparing the re-infection rate between tantalum and the control group after rTHA in septic cases.

According to a meta-analysis using the random effects model, the rate of PJI following rTHA using Ta implants was 2.9% (95% confidence interval [CI]: 2.2%-3.8%) (Fig. 2). The studies showed high heterogeneity (I^2^ = 58.7, Q/df = 2.4). The funnel plot and Egger’s test showed publication bias among the studies (Egger’s *P* = .02, Fig. 3a).

**Figure 4:**
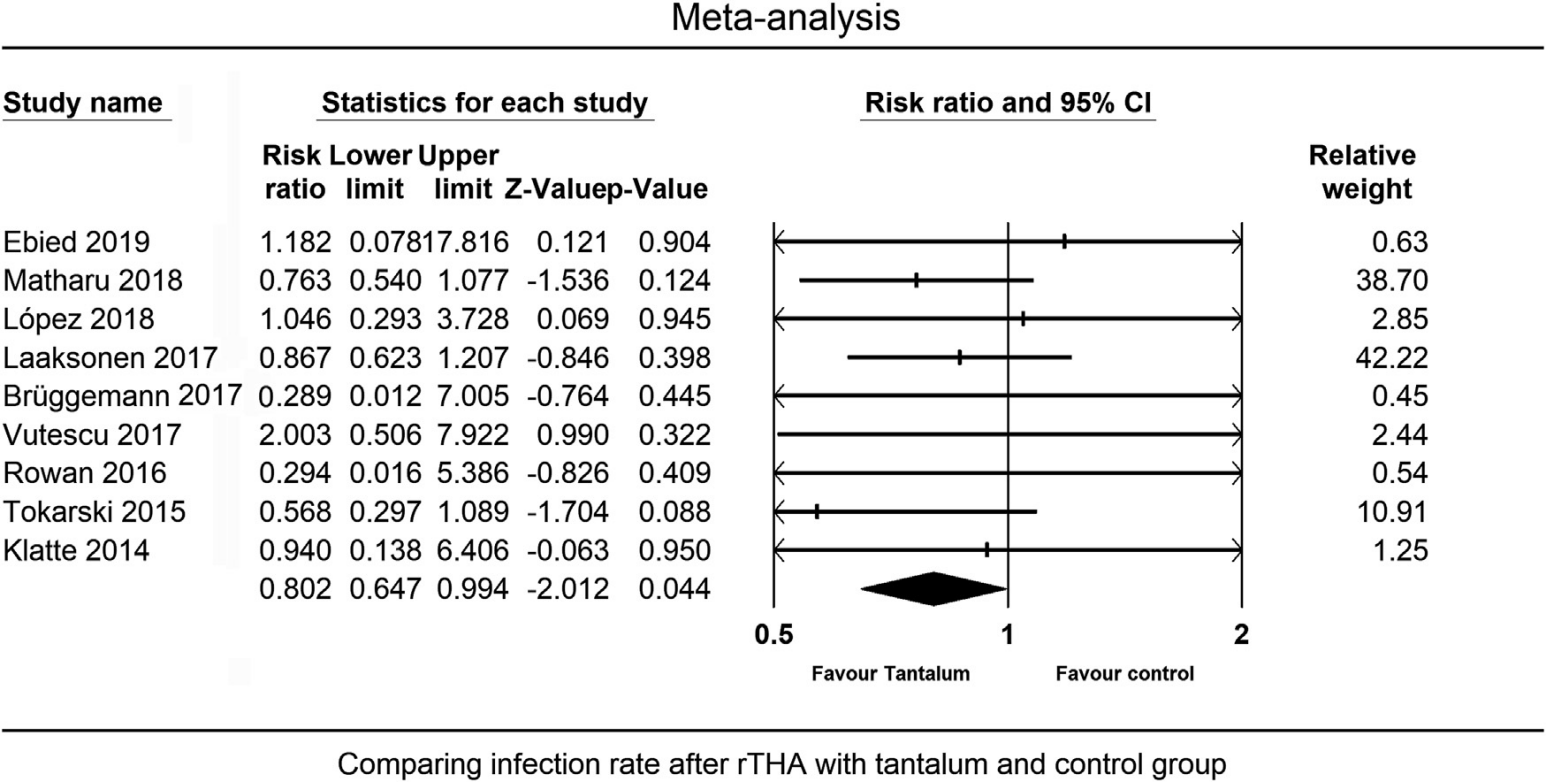
Comparing the infection rate between the Ta and the control group for all-cause rTHA.

Comparing the PJI rate between Ta and control groups (9 studies) revealed a significantly lower rate of PJI in the Ta group with a RR = 0.80 (95% CI = 0.65-0.98, *P* < .05) (Fig. 4). The studies showed low heterogeneity (I^2^ = 0.0, Q/df = 0.5) and no publication bias (Egger’s *P* = .99, [Fig. 3b]). All 9 of these studies were of high quality according to the NOS assessment.

#### Reinfection rate after rTHA in septic cases

**Figure 5:**
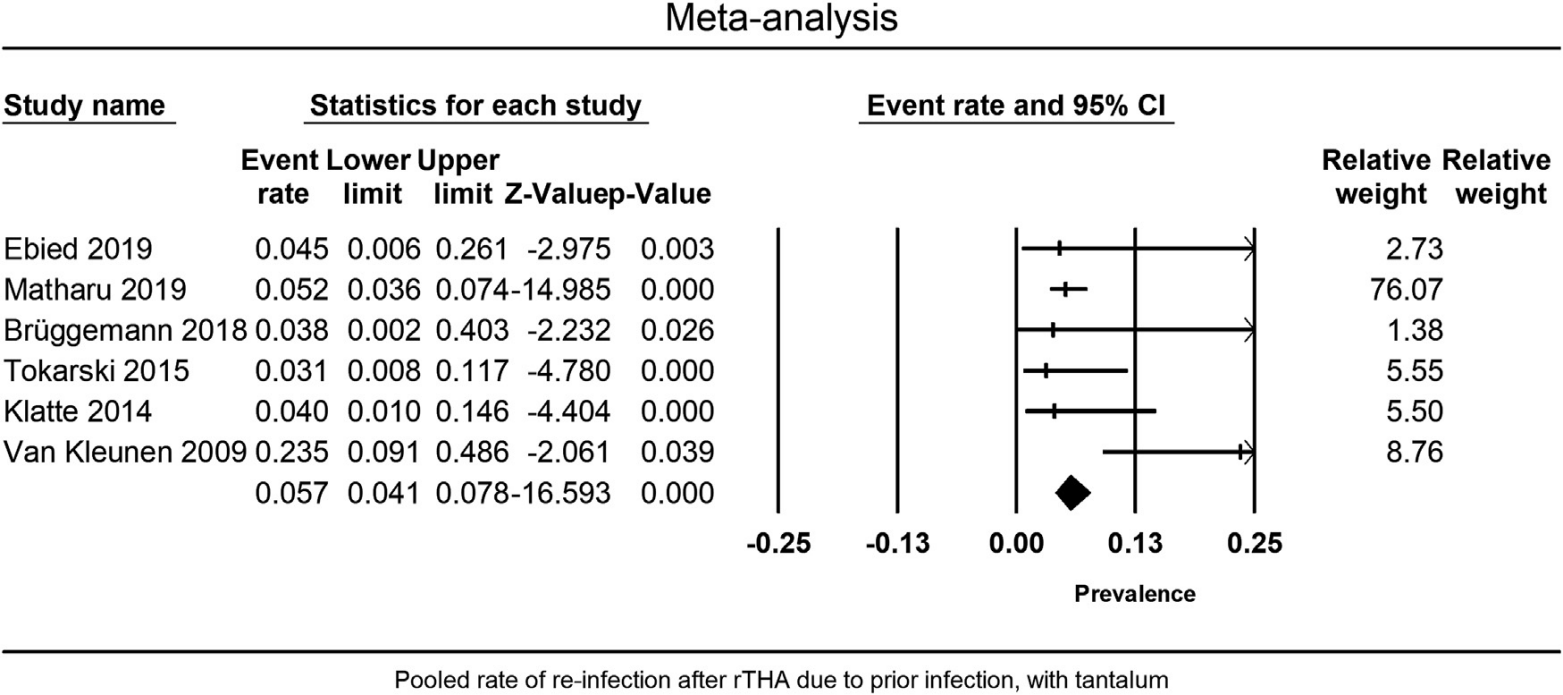
Pooled rate of infection after rTHA with Ta in septic revisions (reinfection).

A meta-analysis of 6 studies showed a 5.7% (95% CI = 4.1%-7.8%) rate of reinfection following rTHA in septic cases (I^2^ = 47.6, Q/df = 1.9) (Fig. 5). The funnel plot showed no publication bias (Egger’s *P* = .86) (Fig. 3c).

**Figure 6:**
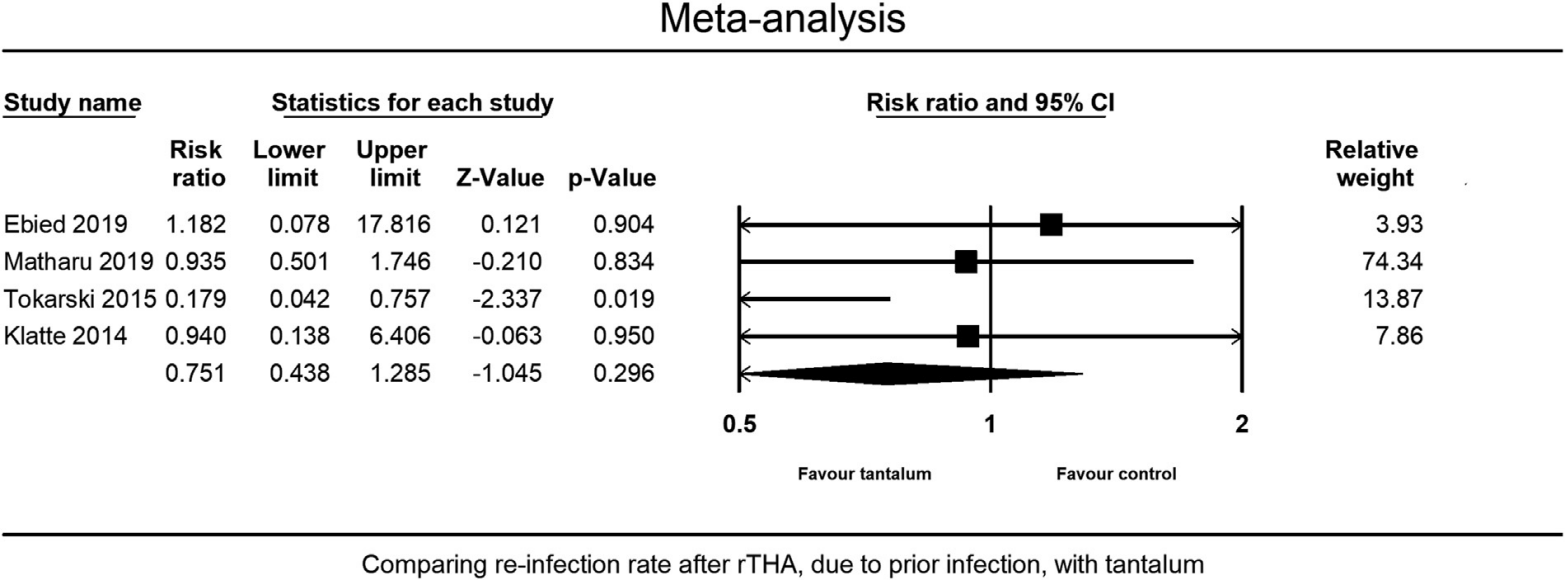
Comparing the reinfection rate between the Ta and the control group for septic rTHA.

There was a trend toward lower reinfection rates following rTHA in septic cases in the Ta group than in the control group (4 studies), although the difference was not statistically significant (RR = 0.75, 95% CI = 0.44-1.29, *P* = .30) (Fig. 6). The studies showed low heterogeneity (I^2^ = 32.3, Q/df = 1.5) and no publication bias (Egger’s *P* = .68) (Fig. 3d). NOS assessed all 4 of these studies as of high quality.

## Discussion

The main finding of this study was that the rate of PJI following rTHA was about 20% lower in the Ta than non-Ta group (RR = 0.80, *P* < .05). When considering only the septic rTHAs, the recurrence rate was similar in both groups (RR = 0.75, 95% CI = 0.44-1.29, *P* = .30). Moreover, the rate of PJI after rTHA using Ta implants was 2.9% overall and 5.7% considering only the septic revisions. In the meta-analysis of the prevalence of PJI after rTHA using Ta (67 studies), significant heterogeneity (I^2^ = 58.7, Q/df = 2.4) and publication bias (Egger’s *P* = .02) were observed. However, when conducting a comparative meta-analysis (Ta vs controls, 9 studies), there was no evidence of publication bias (Egger’s *P* = .99), and heterogeneity was low (I^2^ = 0.0, Q/df = 0.5). The heterogeneity in this analysis can be attributed to various factors, including differences in revision indications (septic or aseptic), Paprosky bone defect classifications, types of Ta implants (augment or cup), as well as variations in population demographics and surgical settings among the 67 studies included.

Tantalum coatings exhibit similar elastic modulus characteristics to trabecular bone. The result is reduced stress shielding, decreased micromotion, enhanced osteointegration, and the prevention of resorption [17]. Studies have reported that Ta augments might possess intrinsic antimicrobial properties and bioactive properties, although there is no consensus [[14], [17],^98^,^99^].

Experimental studies revealed that *S. aureus* adhesion and proliferation on pure Ta are lower than commonly used orthopaedic implant materials [13,14]. Moreover, human leukocytes are more activated and release more cytokines when incubated in Ta-conditioned media [85]. These observations suggest that when leukocytes are activated on the surface of the TM material, a microenvironment is created that may facilitate local host defense [85]. However, the inherent antimicrobial potential of Ta in vivo is uncertain [13,15]. According to Harrison et al, Ta does not have intrinsic antimicrobial or anti-biofilm properties against *S. aureus* and *S. epidermidis* compared to Ti in vitro [15]. Therefore, the reduced rate of PJI associated with Ta use in revision procedures [12] could not be explained by its intrinsic antimicrobial properties [15].

In clinical settings, Tokarski et al found an insignificantly lower infection rate in the Ta cup group than Ti in 990 rTHAs (2.9% vs 5%, *P* = .11) [12]. Considering only the septic revisions, the reinfection rate was significantly lower in the Ta group (3.1% vs 17.5%, *P* = .007) [12]. In a study of septic rTHA cases from the National Joint Registry of England and Wales, Matharu et al found no difference between TM and non-TM implants (5.2% vs 5.6%) [31]. Additionally, TM did not result in a difference in the rate of infection following all-cause rTHA [35]. An earlier study from the same database reported a lower revision rate for infection when TM was used in primary THA (0.5% vs 0.9%, *P* = .001) [70]. A study from Swedish and Australian databases confirms the latter results in all-cause revision [41]. Similarly, a matched case-control study by Klatte et al examined the effect of Ta augments on reinfection in patients with septic single-stage rTHA. At early follow-up, both study groups had a similar reinfection rate of 4% [77]. Hence, further investigations are warranted to answer whether using TM lowers the incidence of infection following rTHA [17].

Some reasons contribute to the possible reduction in infection after rTHA using Ta implants. The literature suggests that Ta may have some antibacterial properties but has not yet reached a clinical consensus [13]. First, Ta is mainly used for severe bone loss and complex reconstructions [86]. In these situations, the surgeon may do a more extensive debridement with wider margins reducing the risk of infection. Second, Ta has a better chance of osseointegration, even in a septic environment, and consequently eliminating the dead spaces [13,87]. The surface morphology of Ta may stimulate host immunity by interacting with leukocytes, releasing specific cytokines, and promoting phagocytosis, thus enhancing the host's natural immunity to infection [85,88]. Another possible mechanism is the 3D structure of Ta, which hinders bacteria from entering and proliferating. This meta-analysis showed a slightly lower postoperative rate of PJI for all-cause rTHA using Ta. However, the PJI rate after rTHA in septic cases did not decrease. As mentioned before, the lower adhesion of Ta to bacterial microorganisms can explain a lower postoperative infection rate after rTHA for Ta. Hypothetically, since the microorganism is already colonized during revision for septic reasons, Ta cannot reduce the risk of postoperative infection.

This study faces several serious limitations. As all the included studies were not level I evidence and most were retrospective, biases may threaten the study's conclusion. However, all 9 comparative studies included in the meta-analysis were of high quality, according to the NOS assessment. The other very considerable problem here is that the weight of the studies is basically overpowered by Matharu 2019 [35] and Laaksonen 2017 [41] papers that have considerable weight in all of the analyses. These are both essentially database studies from the English and Swedish/Australian registries. Although the results showed a significantly lower PJI rate for Ta after all-cause rTHA, the clinical significance is still undecided since the reduction was only 20%. Moreover, the included studies used different criteria to define PJI. Regarding the number of criteria developed in the last decade, inconsistencies exist between the studies regarding PJI diagnosis [[89], [90], [91], [92], [93]]. Furthermore, several studies did not clarify whether infection occurred following septic or aseptic rTHA.

Despite the limitations mentioned, we believe this study is promising and recommend further research. Longitudinal randomized clinical trials evaluating the anti-infection property of Ta in rTHA are recommended. However, the total infection rate is relatively low, and a randomized clinical trials or a cohort study would be challenging.

## Conclusions

This meta-analysis indicates that the rate of PJI following all-cause rTHA is significantly lower when using Ta implants. However, the PJI rate after rTHA did not decrease in septic revisions. Although the results are in favor of Ta, the clinical significance is still unclear since the PJI rate was only reduced by 20%.

## Appendix A. Supplementary data

Supplementary data related to this article can be found at https://doi.org/10.1016/j.artd.2023.101293.

## Conflicts of interest

The authors declare there are no conflicts of interest.

For full disclosure statements refer to https://doi.org/10.1016/j.artd.2023.101293.

## Author contributions

Nasim Eshraghi contributed to methodology, validation, writing – review and editing. SM Javad Mortazavi contributed to conceptualization, supervision, validation, and writing – review and editing. Mohammad Mirahmadi Eraghi contributed to data curation, project administration, validation, and writing – original draft. Fatemeh Kafi contributed to data curation, investigation, visualization, and writing – original draft. Kiarash Roustai-Geraylow contributed to data curation, investigation, and methodology. Alireza Pouramini contributed to data curation, funding acquisition, investigation, and writing – original draft. Erfan Sheikhbahaei contributed to data curation, methodology, and validation. Mohammadreza Razzaghof contributed to conceptualization, validation, and writing – review and editing. Peyman Mirghaderi contributed to conceptualization, data curation, investigation, methodology, software, validation, writing – original draft, and writing – review and editing.

## Figures and Tables

**Supplementary Table:** Quality assessment result based on Newcastle-Ottawa Scale (NOS)

No.	Study author and year	Quality assessment (Newcastle-Ottawa scale (NOS))			Score	Overall quality
		Selection	Comparability	Outcome/Exposure		
1	Russell 2021	∗∗∗	∗	∗∗∗	7	High
2	Cassar-Gheiti 2021	∗∗∗	∗∗	∗∗∗	8	High
3	Xiao 2021	∗∗∗	∗∗	∗∗	7	High
4	Bawale 2021	∗∗	∗	∗∗	5	Medium
5	Simon 2021			∗	1	Low
6	Miettinen 2020	∗∗∗	∗	∗∗∗	7	High
7	Baecker 2020	∗∗	∗	∗∗	5	Medium
8	Chiarlone 2020	∗	∗	∗	3	Low
9	Chacko 2020	∗∗∗	∗	∗∗	6	Medium
10	Zhang 2020	∗	∗∗	∗∗∗	6	Medium
11	Cruz-Pardos 2020	∗∗∗∗	∗∗	∗∗∗	9	High
12	Theil 2019	∗∗∗	∗	∗∗∗	7	High
13	Ebied 2019	∗∗∗∗	∗∗	∗∗∗	9	High
14	Löchel 2019	∗∗∗	∗	∗∗∗	7	High
15	Matharu 2019	∗∗∗∗	∗∗	∗∗∗	9	High
16	Cursaru 2019	∗	∗	∗∗∗	5	Medium
17	Li 2019	∗∗	∗∗	∗∗	6	Medium
18	Brüggemann 2018	∗∗∗	∗∗	∗∗∗	8	High
19	O'Neill 2018	∗∗	∗	∗∗	5	Medium
20	Matharu 2018	∗∗∗∗	∗∗	∗∗∗	9	High
21	Eachempati 2018	∗	∗	∗∗∗	5	Medium
22	Loppini 2018	∗	∗	∗∗∗	5	Medium
23	Chang 2018	∗∗	∗	∗∗	5	Medium
24	Lachiewicz 2018	∗∗∗	∗∗	∗∗∗	8	High
25	López 2018	∗∗∗	∗∗	∗∗	7	High
26	Laaksonen 2017	∗∗∗∗	∗∗	∗∗∗	9	High
27	Prieto 2017	∗	∗∗	∗∗∗	6	Medium
28	Evola 2017	∗∗∗	∗	∗∗∗	7	High
29	Brüggemann 2017	∗∗∗	∗∗	∗∗∗	8	High
30	Jenkins 2017	∗∗	∗	∗∗∗	6	Medium
31	Vutescu 2017	∗∗∗∗	∗∗	∗∗∗	9	High
32	Jeong 2016	∗∗∗	∗∗	∗∗∗	8	High
33	Rowan 2016	∗∗	∗∗	∗∗∗	7	High
34	Flecher 2016	∗∗∗	∗	∗∗	6	Medium
35	Konan 2016	∗	∗∗	∗∗∗	6	Medium
36	Tokarski 2015	∗∗∗∗	∗∗	∗∗∗	9	High
37	Long 2015	∗∗∗∗	∗	∗∗∗	8	High
38	Mohaddes 2015	∗∗∗∗	∗∗	∗∗∗	9	High
39	Whitehouse 2015	∗∗	∗∗	∗∗	6	Medium
40	Callado 2014	∗∗	∗	∗∗	5	Medium
41	Munro 2014	∗∗	∗∗	∗∗∗	7	High
42	Klatte 2014	∗∗∗	∗∗	∗∗∗	8	High
43	Batuyong 2014	∗∗	∗	∗∗∗	6	Medium
44	Moličnik 2014	∗∗	∗	∗∗	5	Medium
45	Abolghasemian 2013	∗∗	∗	∗∗	5	Medium
46	Elganzoury 2013	∗∗∗	∗∗	∗∗	7	High
47	Sporer 2012	∗∗	∗	∗	4	Low
48	Del Gaizo 2012	∗∗	∗	∗	4	Low
49	Borland 2012	∗	∗	∗∗∗	5	Medium
50	Pierannunzii 2011	∗∗∗	∗	∗∗	6	Medium
51	Skyttä 2011	∗∗∗	∗∗	∗∗	7	High
52	Davies 2011	∗∗	∗∗	∗∗	6	Medium
53	Alfaro 2010	∗∗	∗	∗∗	5	Medium
54	Flecher 2010	∗∗∗	∗	∗∗	6	Medium
55	Lachiewicz 2010	∗∗∗	∗	∗∗∗	7	High
56	Fernández-Fairen 2010	∗∗∗	∗∗	∗∗∗	8	High
57	Lakstein 2009	∗	∗	∗∗	4	Low
58	Lingaraj 2009	∗	∗	∗∗	4	Low
59	Siegmeth 2009	∗∗∗	∗	∗	5	Medium
60	Xu 2009	∗∗	∗	∗∗	5	Medium
61	Van Kleunen 2009	∗∗∗∗	∗	∗∗∗	8	High
62	Flecher 2008	∗		∗	2	Low
63	Kim 2008	∗∗∗	∗∗	∗∗∗	8	High
64	Weeden 2007	∗∗∗	∗	∗∗∗	7	High
65	Sporer 2006	∗∗	∗	∗∗	5	Low
66	Sporer 2006	∗∗	∗	∗∗	5	Low
67	Unger 2005	∗∗∗	∗	∗∗	6	Medium

Asterisks indicate scores in each categories.

## Appendix A Supplementary data

Conflict of Interest Statement for Razzaghof

Conflict of Interest Statement for Pouramini

Conflict of Interest Statement for Roustai-Geraylow

Conflict of Interest Statement for Eraghi

Conflict of Interest Statement for Kafi

Conflict of Interest Statement for Javad Mortazavi

Conflict of Interest Statement for Eshraghi

Conflict of Interest Statement for Sheikhbahaei

Conflict of Interest Statement for Mirghaderi
