# Supplementary material for: Does Using Highly Porous Tantalum in Revision Total Hip Arthroplasty Reduce the Rate of Periprosthetic Joint Infection? A Systematic Review and Meta-Analysis
Source: Arthroplast Today. 2024 Jan 17;25:101293. doi: 10.1016/j.artd.2023.101293 (PMC10827600; doi:10.1016/j.artd.2023.101293)
Supplement: Conflict of Interest Statement for Mirghaderi [file mmc9.docx]

***Supplementary file***

**Supplementary Table.** Quality assessment result based on Newcastle-Ottawa Scale (NOS)

| No. | Study author and year | Quality Assessment (Newcastle-Ottawa Scale (NOS)) | | | Score | Overall quality |
| --- | --- | --- | --- | --- | --- | --- |
|  |  | Selection | Comparability | Outcome/Exposure |  |  |
| 1 | Russell 2021 ^127^ | *** | * | *** | 7 | High |
| 2 | Cassar-Gheiti 2021 ^36^ | *** | ** | *** | 8 | High |
| 3 | Xiao 2021 ^33^ | *** | ** | ** | 7 | High |
| 4 | Bawale 2021 ^37^ | ** | * | ** | 5 | Medium |
| 5 | Simon 2021 ^34^ |  |  | * | 1 | Low |
| 6 | Miettinen 2020 ^128^ | *** | * | *** | 7 | High |
| 7 | Baecker 2020 ^38^ | ** | * | ** | 5 | Medium |
| 8 | Chiarlone 2020 ^43^ | * | * | * | 3 | Low |
| 9 | Chacko 2020 ^44^ | *** | * | ** | 6 | Medium |
| 10 | Zhang 2020 ^39^ | * | ** | *** | 6 | Medium |
| 11 | Cruz-Pardos 2020 ^42^ | **** | ** | *** | 9 | High |
| 12 | Theil 2019 ^129^ | *** | * | *** | 7 | High |
| 13 | Ebied 2019 ^50^ | **** | ** | *** | 9 | High |
| 14 | Löchel 2019 ^130^ | *** | * | *** | 7 | High |
| 15 | Matharu 2019 ^46^ | **** | ** | *** | 9 | High |
| 16 | Cursaru 2019 ^131^ | * | * | *** | 5 | Medium |
| 17 | Li 2019 ^49^ | ** | ** | ** | 6 | Medium |
| 18 | Brüggemann 2018 ^54^ | *** | ** | *** | 8 | High |
| 19 | O'Neill 2018 ^45^ | ** | * | ** | 5 | Medium |
| 20 | Matharu 2018 ^96^ | **** | ** | *** | 9 | High |
| 21 | Eachempati 2018 ^51^ | * | * | *** | 5 | Medium |
| 22 | Loppini 2018 ^55^ | * | * | *** | 5 | Medium |
| 23 | Chang 2018 ^57^ | ** | * | ** | 5 | Medium |
| 24 | Lachiewicz 2018 ^86^ | *** | ** | *** | 8 | High |
| 25 | López 2018 ^59^ | *** | ** | ** | 7 | High |
| 26 | Laaksonen 2017 ^26^ | **** | ** | *** | 9 | High |
| 27 | Prieto 2017 ^58^ | * | ** | *** | 6 | Medium |
| 28 | Evola 2017 ^132^ | *** | * | *** | 7 | High |
| 29 | Brüggemann 2017 ^64^ | *** | ** | *** | 8 | High |
| 30 | Jenkins 2017 ^62^ | ** | * | *** | 6 | Medium |
| 31 | Vutescu 2017 ^61^ | **** | ** | *** | 9 | High |
| 32 | Jeong 2016 ^133^ | *** | ** | *** | 8 | High |
| 33 | Rowan 2016 ^68^ | ** | ** | *** | 7 | High |
| 34 | Flecher 2016 ^65^ | *** | * | ** | 6 | Medium |
| 35 | Konan 2016 ^67^ | * | ** | *** | 6 | Medium |
| 36 | Tokarski 2015 ^69^ | **** | ** | *** | 9 | High |
| 37 | Long 2015 ^134^ | **** | * | *** | 8 | High |
| 38 | Mohaddes 2015 ^74^ | **** | ** | *** | 9 | High |
| 39 | Whitehouse 2015 ^77^ | ** | ** | ** | 6 | Medium |
| 40 | Callado 2014 ^71^ | ** | * | ** | 5 | Medium |
| 41 | Munro 2014 ^76^ | ** | ** | *** | 7 | High |
| 42 | Klatte 2014 ^135^ | *** | ** | *** | 8 | High |
| 43 | Batuyong 2014 ^136^ | ** | * | *** | 6 | Medium |
| 44 | Moličnik 2014 ^75^ | ** | * | ** | 5 | Medium |
| 45 | Abolghasemian 2013 ^137^ | ** | * | ** | 5 | Medium |
| 46 | Elganzoury 2013 ^79^ | *** | ** | ** | 7 | High |
| 47 | Sporer 2012 ^138^ | ** | * | * | 4 | Low |
| 48 | Del Gaizo 2012 ^78^ | ** | * | * | 4 | Low |
| 49 | Borland 2012 ^139^ | * | * | *** | 5 | Medium |
| 50 | Pierannunzii 2011 ^140^ | *** | * | ** | 6 | Medium |
| 51 | Skyttä 2011 ^83^ | *** | ** | ** | 7 | High |
| 52 | Davies 2011 ^81^ | ** | ** | ** | 6 | Medium |
| 53 | Alfaro 2010 ^141^ | ** | * | ** | 5 | Medium |
| 54 | Flecher 2010 ^85^ | *** | * | ** | 6 | Medium |
| 55 | Lachiewicz 2010 ^56^ | *** | * | *** | 7 | High |
| 56 | Fernández-Fairen 2010 ^142^ | *** | ** | *** | 8 | High |
| 57 | Lakstein 2009 ^143^ | * | * | ** | 4 | Low |
| 58 | Lingaraj 2009 ^87^ | * | * | ** | 4 | Low |
| 59 | Siegmeth 2009 ^144^ | *** | * | * | 5 | Medium |
| 60 | Xu 2009 ^145^ | ** | * | ** | 5 | Medium |
| 61 | Van Kleunen 2009 ^89^ | **** | * | *** | 8 | High |
| 62 | Flecher 2008 ^98^ | * |  | * | 2 | Low |
| 63 | Kim 2008 ^90^ | *** | ** | *** | 8 | High |
| 64 | Weeden 2007 ^146^ | *** | * | *** | 7 | High |
| 65 | Sporer 2006 ^93^ | ** | * | ** | 5 | Low |
| 66 | Sporer 2006 ^94^ | ** | * | ** | 5 | Low |
| 67 | Unger 2005 ^91^ | *** | * | ** | 6 | Medium |
